# Supplementary material for: Cytosine Methylation in Genomic DNA and Characterization of DNA Methylases and Demethylases and Their Expression Profiles in Viroid-Infected Hop Plants (Humulus lupulus Var. ‘Celeia’)
Source: Cells. 2022 Aug 19;11(16):2592. doi: 10.3390/cells11162592 (PMC9406385; doi:10.3390/cells11162592)
Supplement: Supplementary file 1 [file cells-11-02592-s001.zip › cells-1816841 supplementary figures.pdf]

Supplementary Figures

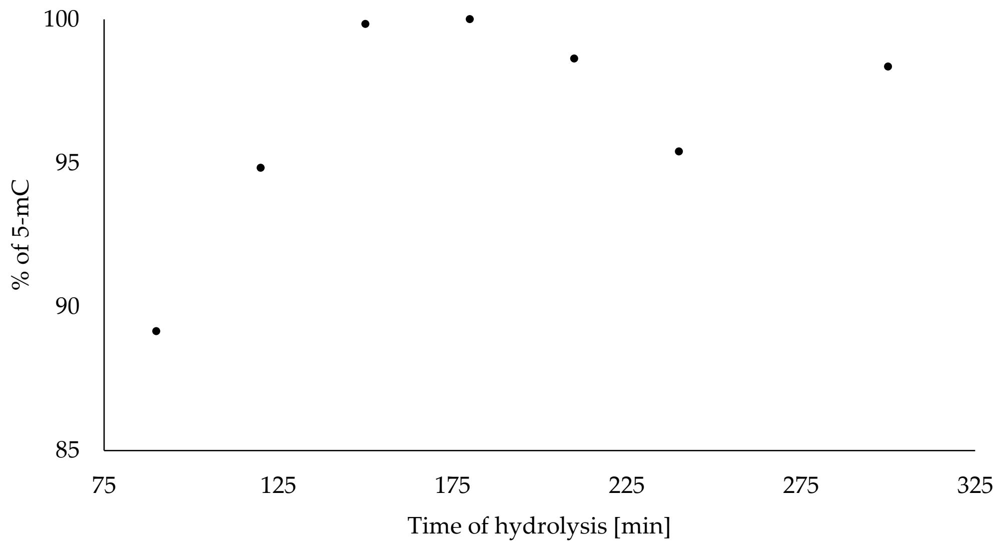

Supplementary Figure S1: Percentage of 5-methylcytosine relative to that of 180 minutes of hydrolysis time.

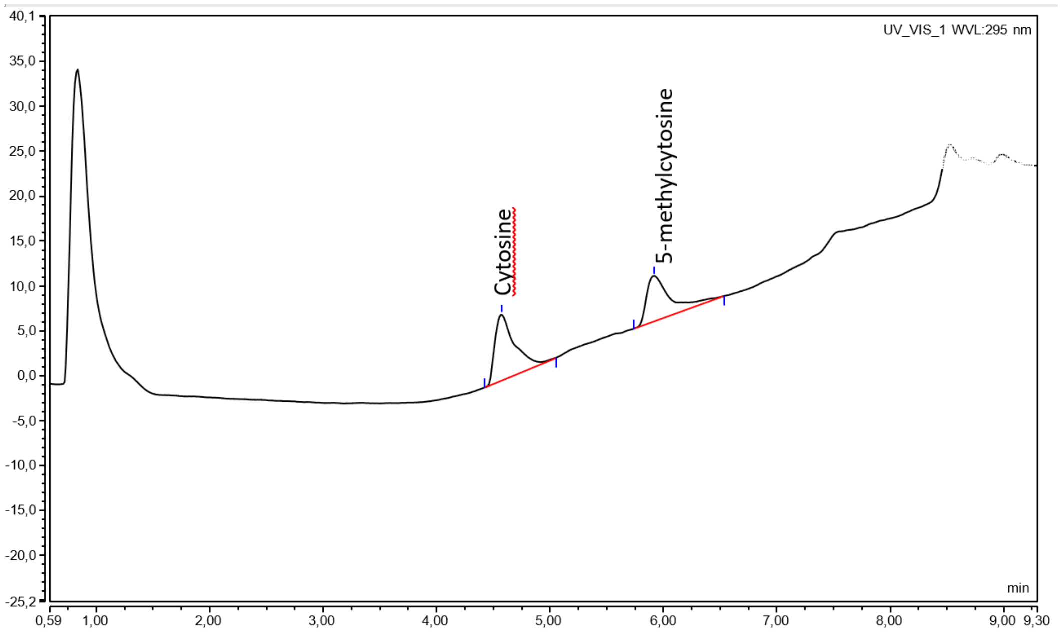

Supplementary Figure S2: HPLC chromatogram of the hydrolysed DNA samples. Two distinct peaks belonging to cytosine and 5-methylcytosine are shown.

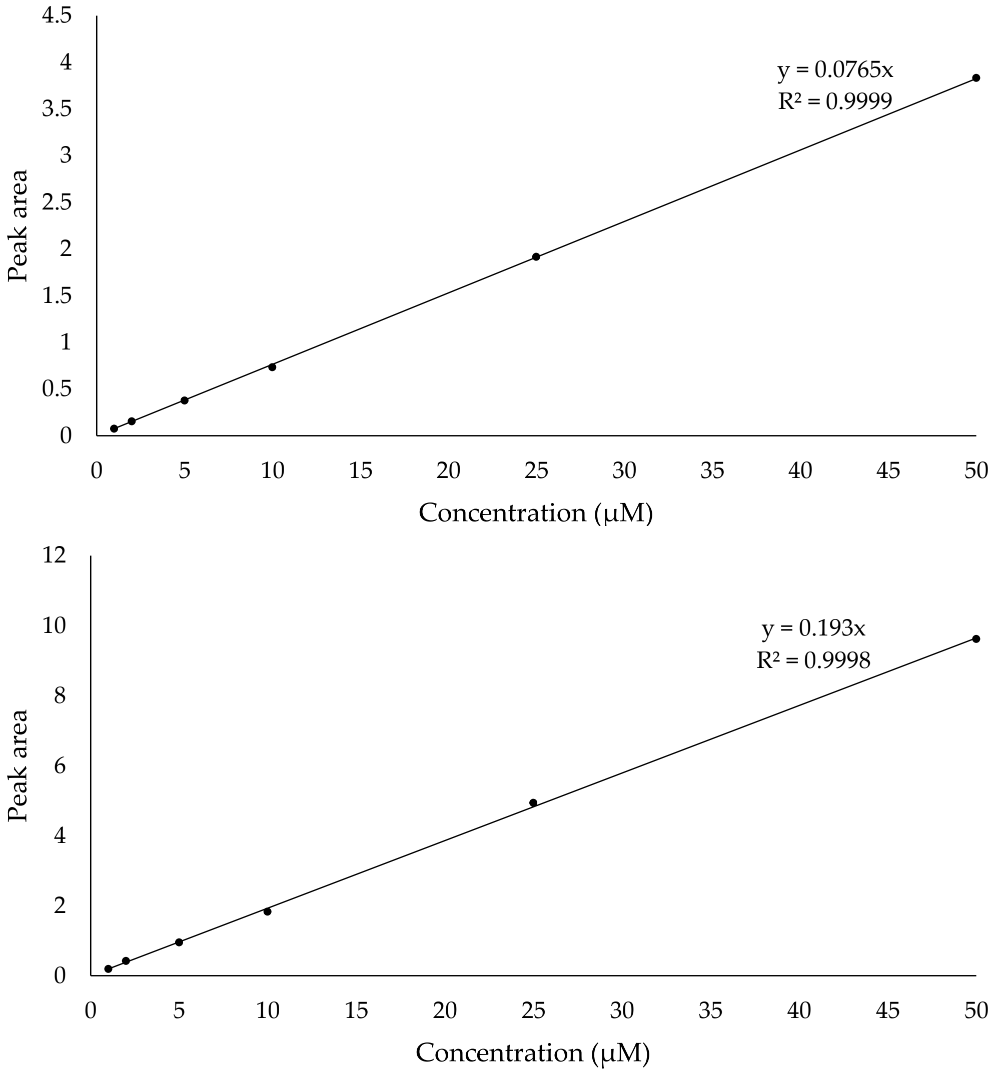

Supplementary Figure S3. Linearity of the HPLC method (Figure A-cytosine and Figure B-methyl-cytosine).
